# Health Disparities in Management of Keratinocyte Carcinoma: A Multicenter Observational Study

**DOI:** 10.3390/cancers18213561

**Published:** 2026-11-06

**Authors:** Margaux Debaveye, Laura Destrooper, Rick Waalboer-Spuij, Annick Meertens, Isabelle Hoorens

**Affiliations:** 1 Department of Dermatology, Ghent University Hospital, B-9000 Ghent, Belgium; 2 Department of Dermatology, Erasmus MC University Medical Center, 3015GD Rotterdam, The Netherlands; 3 Skin Cancer Research Institute Ghent (Skin-CRIG), B-9000 Ghent, Belgium; 4 Department of Public Health and Primary Care, Interuniversity Centre for Health Economics Research (I-CHER), Ghent University, B-9000 Ghent, Belgium

**Keywords:** keratinocyte carcinoma, basal cell carcinoma, cutaneous squamous cell carcinoma, health disparities, Mohs micrographic surgery, educational level, age, multinomial logistic regression

## Abstract

**Background/Objectives:** Keratinocyte carcinoma (KC) treatment is guided primarily by tumor characteristics, but sociodemographic factors may also influence care. We examined associations of age, sex, marital status, and educational level with treatment selection. **Methods:** Adults with actinic keratosis or KC were recruited in four Belgian and Dutch settings between 2020 and September 2023. Treatment was categorized as topical/non-invasive therapy, Mohs micrographic surgery (MMS), or standard surgical excision (reference). Multinomial regression adjusted for histologic group, tumor size, location, recruiting center, and an age-independent Charlson comorbidity component. The primary analysis included 681 participants, with additional sensitivity analyses. **Results:** The model converged normally. Age (global *p* = 0.023) and educational level (*p* = 0.002) were associated with treatment selection; sex and marital status were not. Each additional decade of age was associated with lower odds of MMS versus standard excision (adjusted odds ratio [aOR] 0.755, 95% confidence interval [CI] 0.595–0.959). Low versus high education was associated with lower odds of topical/non-invasive treatment versus standard excision (aOR 0.123, 95% CI 0.034–0.450), but not with MMS (aOR 0.533, 95% CI 0.232–1.222). **Conclusions:** Age and educational level were associated with KC treatment selection after clinical and center adjustment. These associations do not establish inequitable care because relevant patient-, clinician-, and pathway-level determinants were not captured.

## Simple Summary

Keratinocyte carcinomas can be treated with topical or other non-invasive therapies, standard surgical excision, or Mohs micrographic surgery. We examined whether age, sex, marital status, and educational level were associated with treatment selection after accounting for tumor characteristics, comorbidity, and recruiting center. The primary analysis included 681 participants and converged without numerical instability. Older age was associated with lower odds of Mohs surgery rather than standard excision. Participants with low educational attainment had lower odds of topical or non-invasive treatment rather than standard excision, but not of Mohs surgery after center adjustment. Sex and marital status were not independently associated with treatment. These findings describe associations with treatment selection; they do not establish unequal care because patient preference, frailty, referral pathways, clinician recommendation, and treatment availability were not fully measured.

## 1. Introduction

Basal cell carcinoma (BCC) and cutaneous squamous cell carcinoma (cSCC) are commonly grouped under the term keratinocyte carcinoma (KC), reflecting their shared origin from epidermal keratinocytes [1]. KC represents a major and increasing healthcare burden in predominantly fair-skinned populations. Population-based studies have documented rising incidence, and global projections indicate that the burden among older adults will continue to increase as populations age and grow [2,3,4]. Although disease-specific mortality is generally low, KC may lead to repeated procedures, functional or cosmetic impairment, and measurable effects on health-related quality of life [5].

Management includes standard surgical excision, Mohs micrographic surgery (MMS), curettage, cryotherapy, topical agents such as imiquimod and 5-fluorouracil, photodynamic therapy, laser-based treatment, radiotherapy, and, in selected patients, active surveillance [6,7,8]. Vertical tumor depth may also inform treatment planning and surgical strategy. A recent prospective pilot study using portable 20 MHz high-frequency ultrasound demonstrated agreement between ultrasound-estimated and histopathologic BCC depth, suggesting a potential role for non-invasive preoperative depth assessment, although validation in larger cohorts is required [9]. Treatment selection is influenced by histologic subtype, recurrence risk, tumor diameter, anatomic site, patient characteristics, and treatment goals [10,11,12]. Comparative studies demonstrate that treatment options differ in long-term tumor control, procedural burden, tissue preservation, and cosmesis [13,14,15,16,17,18].

Variation in treatment is therefore expected and may represent clinically appropriate personalization. However, it may also reflect health disparities. Health disparities are systematic differences in health, access, or care associated with social, economic, geographic, or demographic disadvantage; not every observed difference is avoidable or unjust [19,20]. European studies have documented variation in skin-cancer pathways and treatment access [21]. Studies from the United States have linked rural residence, race and ethnicity, income, educational level, insurance status, transportation, and cost to barriers in dermatologic care [22,23]. Socioeconomic differences have also been reported among patients receiving MMS and in KC incidence patterns [24,25].

Age, sex, educational level, and marital status represent complementary demographic and social dimensions potentially relevant to treatment selection. Educational level may reflect aspects of socioeconomic position and health literacy, whereas marital status may serve as a limited proxy for household or social support. Age may also reflect frailty, comorbidity, life expectancy, and treatment preferences, all of which may influence the balance between treatment burden and expected benefit [26,27]. Shared decision-making is particularly important when several clinically reasonable options are available [28].

Most previous work has focused on KC incidence, disease burden, access to specialists, or treatment delay rather than on the treatment selected after a patient has entered clinical care. Treatment modalities also differ in long-term recurrence and cost [29,30], making the treatment-decision stage clinically and economically relevant. The specific contribution of this multicenter study is the evaluation of treatment selection in European academic and community settings, with adjustment for measurable tumor characteristics, comorbidity, and recruiting center. We aimed to identify and quantify associations between age, sex, marital status, educational level, and KC treatment selection. We hypothesized that one or more sociodemographic factors would remain associated with treatment after clinical and center adjustment.

## 2. Materials and Methods

### 2.1. Study Design and Setting

This multicenter observational study enrolled adults between 2020 and September 2023 in Belgium and the Netherlands. Belgian participants were recruited at the Department of Dermatology of Ghent University Hospital, Dermatologie Maldegem, a private dermatology practice in Maldegem, and a participating general medical practice in Ghent. Dutch participants were recruited at the Department of Dermatology of Erasmus MC University Medical Center Rotterdam. At Erasmus MC, patients were referred by a general practitioner or specialist, and MMS was performed by dermatologists certified in Mohs surgery. Participants were recruited within these clinical settings; the specific type of consultation at which recruitment occurred was not captured in a harmonized variable. Common eligibility criteria and harmonized variable definitions were used within the same REDCap study framework.

At Ghent University Hospital, patients accessed specialist dermatologic care following referral by a general practitioner or another dermatologist. Initial dermatologic assessment was performed by a dermatology resident and subsequently reviewed by a staff dermatologist. Standard excisions were performed by dermatologic surgeons, including supervised residents and staff dermatologists. MMS was performed by a dedicated dermatologic surgeon. For extensive defects requiring complex reconstruction, care was coordinated with the Department of Plastic and Reconstructive Surgery. Patients attending the private dermatology practice in Maldegem or the participating general practice in Ghent could schedule appointments directly. Detailed pathway-level information, including standard-excision and reconstructive pathways at Erasmus MC, was not systematically harmonized across all participating centers; therefore, site-specific pathways are described only where verified information was available. Recruiting center was included in the primary model to account for differences in referral pathways, treatment availability, and local organization of care.

The study was registered at ClinicalTrials.gov (NCT04814953) and is reported in accordance with the STROBE statement [31].

### 2.2. Participants and Unit of Analysis

Adults were eligible if they had at least one actinic keratosis (AK) or KC diagnosed by a dermatologist or confirmed histopathologically within the preceding 10 years. Patients with a personal history of melanoma or another non-KC skin malignancy, such as Merkel cell carcinoma, were excluded. All participants provided written informed consent. The KC analyses were restricted to participants with BCC, cSCC, or both.

The unit of analysis was the individual participant rather than the individual lesion. For participants with multiple tumors, tumor and treatment variables were collapsed into participant-level categories using predefined hierarchical rules. If both BCC and cSCC were present, cSCC subtype and cSCC size took precedence over the corresponding BCC characteristics. Tumor size was dichotomized as <1 cm versus ≥1 cm; the three observations in the original >6 cm category were included in the ≥1 cm group. Location was the participant-level facial versus non-facial variable recorded in the study database.

### 2.3. Data Collection and Variable Definitions

Demographic, clinical, tumor, and treatment data were entered by the treating clinician and managed using REDCap electronic data-capture tools hosted at Ghent University Hospital. The sociodemographic variables of interest were age, sex, marital status, and educational level. These variables were selected a priori because they were consistently collected using harmonized definitions across all participating settings and represented complementary demographic and social dimensions potentially relevant to treatment selection. Employment status, occupation, postcode-level deprivation, income, travel distance, caregiving responsibilities, and family logistics were not consistently collected and therefore could not be analyzed. Sex was recorded as female or male. Marital status was reduced to married/cohabiting versus single/widowed. Educational level was grouped as low (no formal diploma or primary education), secondary, high (higher education or university degree), and other. High education served as the reference category.

Because diagnostic category and histologic subtype encoded overlapping information, diagnostic category was not included in the primary analysis. Histologic subtype was grouped into four clinically interpretable categories: nodular BCC (reference); superficial or in-situ disease (superficial BCC or cSCC in situ); aggressive BCC (morpheaform, sclerodermiform, micronodular, or infiltrating); and invasive cSCC (well-differentiated, poorly differentiated, undifferentiated, or lymphatic/metastatic disease). This grouping was selected to reduce sparse cells while preserving clinically relevant distinctions. Tumor depth was not systematically available and could not be included.

The dependent variable was treatment category. Topical and non-invasive treatment comprised imiquimod, 5-fluorouracil, laser therapy, cryotherapy, and photodynamic therapy. Surgical categories were MMS and standard surgical excision. For participants with more than one recorded treatment, surgery took precedence over topical/non-invasive treatment. In the single participant who underwent both MMS and standard excision, MMS took precedence. These rules produced three mutually exclusive analytic outcome categories.

The database contained an age-adjusted Charlson Comorbidity Index. Because age was a predictor of primary interest, the standard age component was subtracted and an age-independent comorbidity component was included in the primary model to avoid adjusting for age twice. A sensitivity analysis used the recorded number of comorbidities instead.

### 2.4. Statistical Analysis

Continuous and categorical variables were summarized using means with standard deviations and counts with percentages, respectively. Participant flow and missingness were reported for the full cohort, the KC-eligible cohort, the complete-case cohort based on the candidate model variables, and the primary analytic cohort. The 693 complete cases were compared with the 69 KC-eligible participants who had at least one missing candidate model variable to assess potential selection bias.

The primary multinomial logistic regression used standard surgical excision as the reference outcome. Age was scaled per 10-year increase. The model included age, sex, marital status, educational level, histologic group, tumor size, tumor location, recruiting center, and the age-independent Charlson comorbidity component. The heterogeneous “other education” category (*n* = 12) was excluded from the primary analysis, yielding 681 participants, and was restored in a sensitivity analysis. Nodular BCC, tumors ≥ 1 cm, non-facial location, Ghent University Hospital, high education, male sex, and single/widowed status served as reference categories.

Results are reported as adjusted odds ratios (aORs), 95% confidence intervals (CIs), and two-sided *p* values. Overall predictor effects were evaluated using likelihood-ratio tests comparing the full model with a reduced model omitting the corresponding predictor block. Model fit was described using the likelihood-ratio χ^2^ statistic and McFadden pseudo-R^2^. Convergence, standard errors, and coefficient magnitude were inspected to assess numerical stability. A quadratic age term was tested to assess departure from linearity.

Sensitivity analyses (i) restored the “other education” category; (ii) replaced center with country; (iii) used diagnostic category instead of histologic group; (iv) retained participants with missing clinical covariates by adding explicit missing categories; and (v) replaced the age-independent Charlson component with the recorded number of comorbidities. Because the simplified primary model converged without evidence of separation, robustness was assessed through multiple alternative model specifications rather than penalized regression. Data management and initial variable derivation were performed in IBM SPSS Statistics, version 31. Multinomial regression modeling and sensitivity analyses were conducted in Python 3.13 using statsmodels version 0.14.6. OpenAI ChatGPT Pro version was used to assist with analysis-code development and manuscript preparation; all code, outputs, references, interpretations, and text were reviewed and verified by the authors. Statistical significance was defined as *p* < 0.05.

### 2.5. Ethical Considerations

The study was conducted in accordance with the Declaration of Helsinki and approved by the Ethics Committee of Ghent University Hospital (EC/2019/1352; Belgian registration number B670201941520; approval date: 18 October 2019). Written informed consent was obtained from all participants.

## 3. Results

### 3.1. Participant Flow and Cohort Characteristics

**Figure 1:**
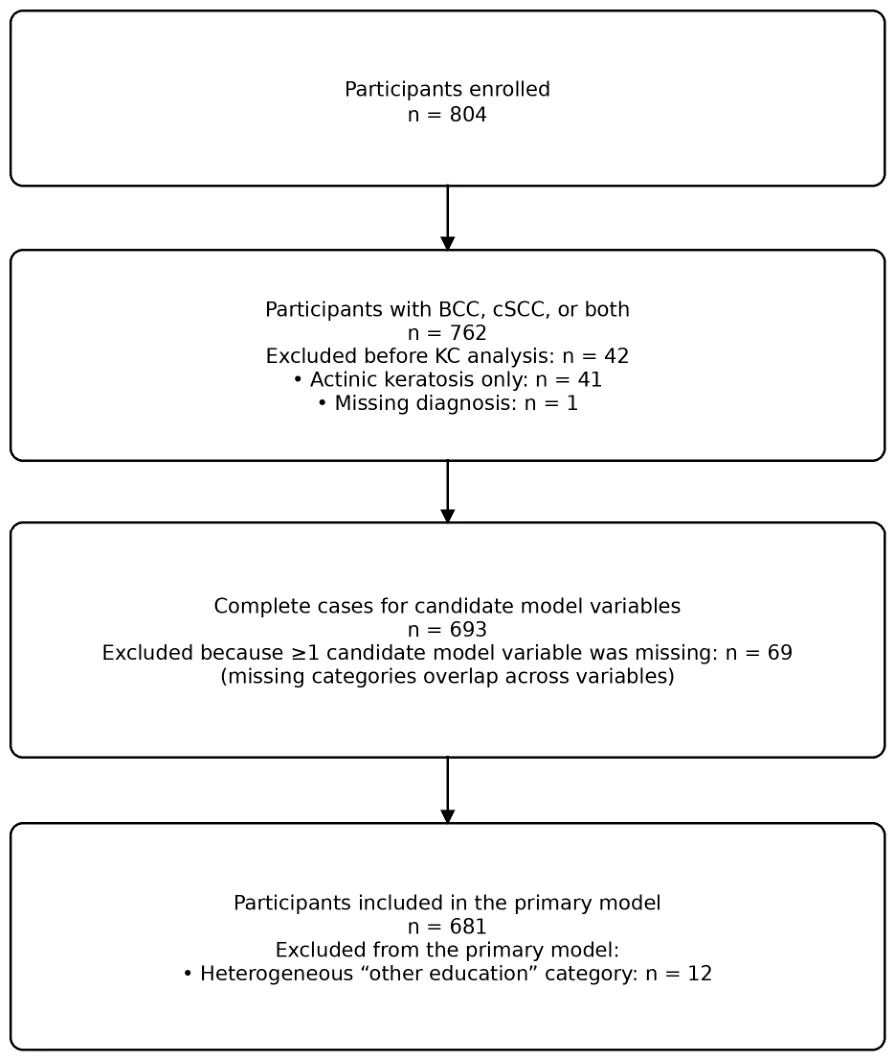
Participant flow from enrollment to the primary multinomial regression analysis.

A total of 804 participants were enrolled: 692 (86.1%) in Belgium and 112 (13.9%) in the Netherlands. Forty-one participants had AK only and one had a missing diagnostic category, leaving 762 participants with BCC, cSCC, or both. Sixty-nine KC-eligible participants had at least one missing candidate model variable, leaving 693 complete cases. Excluding the 12 participants in the heterogeneous “other education” category yielded 681 participants for the primary model (Figure 1).

The mean age of the full cohort was 66.7 years (SD 11.4), and 376 participants (46.8%) were female. Recruitment included university, private dermatology, and general-practice settings. Baseline characteristics are summarized in Table 1.

### 3.2. Missing Data and Comparison of Analytic and Excluded KC Participants

Among the 69 KC-eligible participants excluded from the complete-case cohort, missing values occurred for tumor size (*n* = 38), histologic subtype (*n* = 26), location (*n* = 18), treatment category (*n* = 7), education (*n* = 4), and marital status (*n* = 3); categories overlap because some participants had more than one missing variable. Age, sex, and diagnostic category were complete in this group. Complete and incomplete KC participants were similar in age, sex, marital status, educational level, and known treatment distribution. Incomplete cases were more frequently recruited outside Ghent University Hospital and more often had cSCC or combined BCC/cSCC, indicating that missingness was not completely random (Supplementary Tables S1 and S2).

### 3.3. Primary Model

The primary model included 681 participants: 138 (20.3%) in the topical/non-invasive category, 250 (36.7%) in the MMS category, and 293 (43.0%) in the standard-excision category. It converged normally without a Hessian warning, zero standard errors, or extreme non-estimable coefficients. The model significantly improved fit compared with the intercept-only model (likelihood-ratio χ^2^ = 613.65; 28 df; *p* < 0.001) and had a McFadden pseudo-R^2^ of 0.427.

### 3.4. Sociodemographic Factors and Treatment Selection

Age was associated with treatment selection overall (global *p* = 0.023). Each additional decade of age was associated with lower odds of MMS rather than standard excision (aOR 0.755, 95% CI 0.595–0.959; *p* = 0.021), corresponding to approximately 24.5% lower relative odds per decade. The association between age and topical/non-invasive treatment versus standard excision did not reach statistical significance (aOR 0.810, 95% CI 0.641–1.025; *p* = 0.080). There was no evidence of a non-linear age effect (quadratic-term likelihood-ratio *p* = 0.618).

Educational level was associated with treatment selection overall (global *p* = 0.002). Compared with participants with high educational attainment, those with low educational attainment had lower odds of topical/non-invasive treatment rather than standard excision (aOR 0.123, 95% CI 0.034–0.450; *p* = 0.002). Low educational attainment was not independently associated with MMS rather than standard excision after center and comorbidity adjustment (aOR 0.533, 95% CI 0.232–1.222; *p* = 0.137). Secondary and high education did not differ for either treatment contrast. Sex (global *p* = 0.063) and marital status (global *p* = 0.967) were not independently associated with treatment selection. The estimate for female sex and MMS was above one but remained imprecise and non-significant (aOR 1.662, 95% CI 0.990–2.788; *p* = 0.054) (Table 2).

### 3.5. Clinical and Contextual Factors

Histologic group, tumor size, tumor location, and recruiting center were strongly associated with treatment selection (all global *p* < 0.001). Compared with nodular BCC, superficial BCC or cSCC in situ was more often managed with topical/non-invasive treatment rather than standard excision and less often with MMS. Aggressive BCC was more likely to be managed with MMS than standard excision, whereas invasive cSCC was less likely to receive MMS than nodular BCC. Tumors < 1 cm had lower odds of either topical/non-invasive treatment or MMS relative to standard excision. Facial location was very strongly associated with MMS rather than standard excision (aOR 38.707, 95% CI 16.711–89.655; *p* < 0.001).

Recruiting center was independently associated with treatment selection (global *p* < 0.001), consistent with differences in referral pathways and local treatment availability. The age-independent Charlson comorbidity component was not significantly associated with treatment (global *p* = 0.358). These center effects reinforce the importance of interpreting demographic associations within the organization of care rather than as direct evidence of inequity (Table 3).

### 3.6. Sensitivity Analyses

The principal conclusions were stable across alternative specifications (Supplementary Table S3). Restoring the “other education” group (*n* = 693), replacing center with country (*n* = 681), using diagnostic category instead of histologic group (*n* = 696), retaining missing clinical values as explicit categories (*n* = 735), and replacing the Charlson component with the number of comorbidities (*n* = 681) all preserved a strong association between low education and lower odds of topical/non-invasive treatment versus standard excision. None showed a statistically significant low-versus-high education association for MMS versus standard excision. The lower odds of MMS with increasing age were retained in most specifications. When the “other education” group was restored, its MMS estimate was large but highly imprecise (aOR 8.593, 95% CI 1.311–56.337), confirming that this small, heterogeneous category should not drive the primary interpretation.

## 4. Discussion

### 4.1. Principal Findings

In this multicenter Belgian–Dutch cohort, age and educational level were associated with KC treatment selection after adjustment for tumor characteristics, recruiting center, and comorbidity. Older age was consistently associated with lower odds of MMS rather than standard excision. Low educational attainment was associated with lower odds of topical/non-invasive treatment rather than standard excision. After removal of the overlapping diagnosis variable and adjustment for center, low educational attainment was not independently associated with MMS. Sex and marital status were not significant independent predictors. The model converged normally and the main findings were broadly consistent across sensitivity analyses.

### 4.2. Clinical Context and Treatment Variation

The strong associations with histologic group, tumor size, facial location, and recruiting center emphasize that treatment selection is clinically and organizationally structured. Margin-controlled, tissue-sparing surgery is particularly relevant for high-risk or anatomically critical tumors [6,7,8,11,12,13,14], whereas topical and other non-invasive approaches are generally reserved for selected low-risk lesions [15,16,17,32]. Long-term recurrence and cost also differ across options [14,29,30]. Tumor depth may provide additional information for surgical planning, but it was not systematically available in this dataset [9].

The strong center effect is clinically plausible because the participating sites differed in referral route, setting, and local availability of MMS. Belgium and the Netherlands both provide access to specialist dermatologic care and participants were studied using common eligibility criteria and harmonized data definitions, but the clinical settings should not be assumed to be identical. Center adjustment therefore improves interpretation but cannot fully capture unmeasured differences in triage, waiting time, operator expertise, or treatment capacity.

### 4.3. Age

The adjusted estimate corresponded to approximately 24.5% lower odds of MMS rather than standard excision per additional decade of age. This association may reflect frailty, life expectancy, wound-healing considerations, procedural burden, travel requirements, caregiver support, or treatment preference [26,27]. Clinicians may appropriately tailor treatment to patient goals and expected time to benefit. Conversely, age may influence referral to an MMS center or the range of options discussed. Because functional status, frailty, life expectancy, clinician recommendation, and patient preference were not directly measured, the present study cannot distinguish appropriate personalization from unwarranted age-related variation. Shared decision-making and standardized information remain important when multiple reasonable treatments are available [28].

### 4.4. Educational Level

Educational level may capture differences in health literacy, communication, navigation of referral systems, awareness of specialist options, occupational flexibility, and preferences regarding recurrence risk, cosmetic outcome, or procedural burden. Although direct financial barriers may be attenuated in predominantly publicly financed systems, opportunity costs, information, geography, and access to specialist pathways can still differ [19,20,21,22,23,24,25].

After adjustment for center and use of a simplified clinical model, the association with education was concentrated in the comparison between topical/non-invasive treatment and standard excision. The low-versus-high education association for MMS was attenuated and no longer statistically significant. This distinction is important: the findings do not support a broad conclusion that patients with low educational attainment were systematically less likely to receive MMS. They instead identify a more specific treatment-selection pattern that warrants investigation in data containing patient preferences, options offered, referral history, and treatment availability.

### 4.5. Sex, Marital Status, and Other Social Determinants

Neither sex nor marital status was independently associated with treatment selection. The estimate for female sex and MMS was close to the conventional significance threshold, so smaller sex differences cannot be excluded. Marital status was clearly null in the primary model. Nevertheless, marital status is an incomplete measure of social support. Occupation, employment flexibility, income, area-level deprivation, travel distance, transport access, caregiving responsibilities, family logistics, and health literacy were unavailable or insufficiently harmonized. Future studies should capture these dimensions directly rather than relying on education or partnership status as proxies.

### 4.6. Strengths and Limitations

Strengths include the multicenter Belgian–Dutch setting, recruitment across university and community practice, a relatively large cohort, inclusion of several commonly used treatment modalities, adjustment for major tumor characteristics and center, and multiple sensitivity analyses. The primary model avoided simultaneous inclusion of overlapping diagnosis and subtype variables, simplified sparse histologic categories, selected a common reference group, and excluded the heterogeneous “other education” category from the primary analysis. It converged without a Hessian warning or non-estimable coefficients.

Several limitations remain. First, the participant-level unit of analysis and hierarchical coding may obscure lesion-specific decision-making in patients with multiple tumors; tumor characteristics cannot always be unequivocally linked to the lesion that generated the treatment category. Second, 69 KC-eligible participants had incomplete candidate-model data. They were broadly similar demographically to complete cases, but differed by recruiting center and diagnosis, so selection bias remains possible. Third, center fixed effects account for measured site membership but not all pathway-level differences or within-center clustering. Fourth, tumor depth was unavailable. Fifth, frailty, functional status, life expectancy, occupation, income, postcode, travel burden, health literacy, clinician recommendation, referral pathway, treatment availability, and patient preference were not fully captured. Sixth, the “other education” category was small and heterogeneous; its estimate was unstable when restored. Finally, the observational design supports association rather than causation and cannot determine whether treatment differences were avoidable, unjust, patient-preferred, or clinically appropriate.

## 5. Conclusions

In this multicenter cohort, age and educational level were associated with KC treatment selection after adjustment for measured tumor characteristics, recruiting center, and comorbidity, whereas sex and marital status were not. Older age was associated with lower odds of MMS rather than standard excision. Low educational attainment was associated with lower odds of topical/non-invasive treatment rather than standard excision, but not with MMS after center adjustment. Clinical and center factors were major determinants of treatment.

These findings identify associations with treatment selection and do not constitute direct evidence of inequitable care. Patient preference, frailty, clinician recommendation, referral pathways, treatment availability, travel burden, and other social determinants may account for part or all of the observed variation. Future lesion-level studies that document the options offered and the decision-making pathway are needed to distinguish appropriate personalized care from potentially avoidable disparity.

## Figures and Tables

**Table 1:** Demographic and clinical characteristics of the enrolled cohort (*N* = 804).

Characteristic	Value, *n* (%) or Mean (SD)
**Recruiting center**	
Ghent University Hospital	527 (65.5%)
Dermatologie Maldegem	140 (17.4%)
Erasmus MC University Medical Center	112 (13.9%)
Participating general practice in Ghent	25 (3.1%)
**Age and sex**	
Age, years	66.7 (11.4)
Female	376 (46.8%)
Male	428 (53.2%)
**Educational level**	
No formal degree	25 (3.1%)
Primary school	54 (6.7%)
Secondary school	276 (34.3%)
Higher education	274 (34.1%)
University degree	157 (19.5%)
Other	13 (1.6%)
Missing	5 (0.6%)
**Marital status**	
Married	547 (68.0%)
Single	115 (14.3%)
Cohabiting with partner or others	91 (11.3%)
Widowed	48 (6.0%)
Missing	3 (0.4%)
**Recorded diagnosis/history**	
Actinic keratosis only	41 (5.1%)
Single BCC	292 (36.3%)
Multiple BCCs	245 (30.5%)
Single cSCC	63 (7.8%)
Multiple cSCCs	33 (4.1%)
BCC and cSCC	129 (16.0%)
Missing	1 (0.1%)
**Last recorded treatment category**	
Standard surgical excision	306 (38.1%)
Mohs micrographic surgery	266 (33.1%)
Cryotherapy	24 (3.0%)
Imiquimod	6 (0.7%)
Laser therapy	20 (2.5%)
5-fluorouracil	12 (1.5%)
Photodynamic therapy	43 (5.3%)
Two recorded treatments	70 (8.7%)
Three recorded treatments	8 (1.0%)
None or missing	45 (5.6%)
Other	4 (0.5%)
**Comorbidity**	
Age-adjusted Charlson Comorbidity Index	3.06 (2.12)

BCC, basal cell carcinoma; cSCC, cutaneous squamous cell carcinoma. Treatment categories in this descriptive table are mutually exclusive raw database categories; “two recorded treatments” and “three recorded treatments” indicate exactly two and three recorded treatments, respectively. The regression outcome was subsequently collapsed into three analytic categories.

**Table 2:** Multivariable multinomial logistic regression: sociodemographic predictors of treatment selection (*N* = 681).

Predictor	Comparison	Topical/Non-Invasive vs. Standard Excision, aOR (95% CI)	*p*	MMS vs. Standard Excision, aOR (95% CI)	*p*
Age (global *p* = 0.023)	Per 10-year increase	0.810 (0.641–1.025)	0.080	0.755 (0.595–0.959)	0.021
Sex (global *p* = 0.063)	Female vs. male	0.753 (0.447–1.269)	0.287	1.662 (0.990–2.788)	0.054
Marital status (global *p* = 0.967)	Married/cohabiting vs. single/widowed	1.038 (0.577–1.867)	0.900	1.080 (0.579–2.016)	0.808
Educational level (global *p* = 0.002)	Low vs. high	0.123 (0.034–0.450)	0.002	0.533 (0.232–1.222)	0.137
	Secondary vs. high	1.055 (0.621–1.793)	0.843	1.057 (0.616–1.812)	0.842

Standard surgical excision is the reference outcome. High educational level, male sex, and single/widowed status are predictor reference categories. aOR, adjusted odds ratio; CI, confidence interval; MMS, Mohs micrographic surgery.

**Table 3:** Multivariable multinomial logistic regression: clinical and contextual covariates (*N* = 681).

Covariate	Comparison	Topical/Non-Invasive vs. Standard Excision, aOR (95% CI)	*p*	MMS vs. Standard Excision, aOR (95% CI)	*p*
Histologic group (global *p* < 0.001)	Superficial BCC/cSCC in situ vs. nodular BCC	12.212 (6.130–24.328)	<0.001	0.079 (0.030–0.206)	<0.001
	Aggressive BCC vs. nodular BCC	0.571 (0.119–2.735)	0.483	2.255 (1.220–4.170)	0.010
	Invasive cSCC vs. nodular BCC	2.071 (0.808–5.308)	0.129	0.305 (0.148–0.627)	0.001
Tumor size (global *p* < 0.001)	<1 cm vs. ≥1 cm	0.489 (0.293–0.817)	0.006	0.322 (0.185–0.561)	<0.001
Tumor location (global *p* < 0.001)	Facial vs. non-facial	1.396 (0.834–2.338)	0.204	38.707 (16.711–89.655)	<0.001
Recruiting center (global *p* < 0.001)	Dermatologie Maldegem vs. Ghent University Hospital	2.231 (1.190–4.181)	0.012	0.048 (0.010–0.233)	<0.001
	Erasmus MC vs. Ghent University Hospital	0.964 (0.295–3.154)	0.951	13.181 (5.355–32.444)	<0.001
	General practice in Ghent vs. Ghent University Hospital	1.764 (0.265–11.739)	0.557	4.633 (1.015–21.135)	0.048
Comorbidity (global *p* = 0.358)	Age-independent Charlson component, per point	0.991 (0.843–1.165)	0.915	0.884 (0.746–1.047)	0.152

Standard surgical excision is the reference outcome. Nodular BCC, tumors ≥ 1 cm, non-facial location, and Ghent University Hospital are reference categories. aOR, adjusted odds ratio; BCC, basal cell carcinoma; CI, confidence interval; cSCC, cutaneous squamous cell carcinoma; MMS, Mohs micrographic surgery.

## Data Availability

The data used in this study are available from the corresponding author on reasonable request, subject to applicable ethical and data-protection requirements.

## Supplementary Materials

The following supporting information can be downloaded at: https://www.mdpi.com/article/10.3390/cancers18213561/s1, Table S1: Missing candidate model variables among 69 incomplete KC participants. Counts overlap because a participant could have more than one missing variable; Table S2: Comparison of complete cases and KC-eligible participants excluded because of incomplete candidate-model data; Table S3: Sensitivity analyses for age and low educational attainment; Table S4: Primary model fit and global likelihood-ratio tests (*n* = 681).

## Abbreviations

aOR	Adjusted odds ratio
AK	Actinic keratosis
BCC	Basal cell carcinoma
CI	Confidence interval
cSCC	Cutaneous squamous cell carcinoma
KC	Keratinocyte carcinoma
MMS	Mohs micrographic surgery
REDCap	Research Electronic Data Capture
