# Supplementary material for: Health Disparities in Management of Keratinocyte Carcinoma: A Multicenter Observational Study
Source: Cancers (Basel). 2026 Nov 6;18(21):3561. doi: 10.3390/cancers18213561 (PMC13761536; doi:10.3390/cancers18213561)
Supplement: Supplementary file 1 [file cancers-18-03561-s001.zip › cancers-4600263-supplementary.pdf]

cancers MDPI

Supplementary Materials

Health Disparities in Management of Keratinocyte Carcinoma: A Multicenter Observational Study

Margaux Debaveye, Laura Destrooper, Rick Waalboer-Spuij, Annick Meertens and Isabelle Hoorens

Table S1. Missing candidate model variables among 69 incomplete KC participants. Counts overlap because a participant could have more than one missing variable.

Variable	Participants with missing data, n
Treatment category	7
Age	0
Sex	0
Marital status	3
Educational level	4
Histologic subtype	26
Tumor size	38
Diagnostic category	0
Tumor location	18

Table S2. Comparison of complete cases and KC-eligible participants excluded because of incomplete candidate-model data.

Characteristic	Complete cases (n=693)	Incomplete KC cases (n=69)	p
Age, mean (SD)	66.5 (11.5)	67.7 (12.4)	0.477
Female	325 (46.9%)	38 (55.1%)	0.242
Male	368 (53.1%)	31 (44.9%)	
Low educational level	65 (9.4%)	6 (9.2%)	0.661
Secondary education	235 (33.9%)	27 (41.5%)	
High educational level	381 (55.0%)	31 (47.7%)	
Other education	12 (1.7%)	1 (1.5%)	
Married/cohabiting	548 (79.1%)	52 (78.8%)	1.000
Single/widowed	145 (20.9%)	14 (21.2%)	
BCC	500 (72.2%)	37 (53.6%)	0.003
cSCC	80 (11.5%)	16 (23.2%)	
BCC and cSCC	113 (16.3%)	16 (23.2%)	
Topical/non-invasive	140 (20.2%)	15 (24.2%)	0.341
MMS	254 (36.7%)	17 (27.4%)	
Standard excision	299 (43.1%)	30 (48.4%)	
Ghent University Hospital	490 (70.7%)	28 (40.6%)	<0.001
Dermatologie Maldegem	93 (13.4%)	19 (27.5%)	
Erasmus MC	92 (13.3%)	18 (26.1%)	
General practice in Ghent	18 (2.6%)	4 (5.8%)	

Percentages for education, marital status, and treatment use the number with known data as denominator. p values are descriptive and were obtained using Welch's t test for age and chi-square tests for categorical variables. BCC, basal cell carcinoma; cSCC, cutaneous squamous cell carcinoma; KC, keratinocyte carcinoma; MMS, Mohs micrographic surgery.

Table S3. Sensitivity analyses for age and low educational attainment.

(A) Age associations

Model	n	Topical/non-invasive vs standard excision, aOR (95% CI)	p	MMS vs standard excision, aOR (95% CI)	p
Primary model	681	0.810 (0.641–1.025)	0.080	0.755 (0.595–0.959)	0.021
Including "other" education	693	0.813 (0.643–1.028)	0.083	0.748 (0.590–0.948)	0.017
Country instead of center	681	0.808 (0.642–1.017)	0.069	0.797 (0.637–0.996)	0.046
Diagnosis instead of histologic group	696	0.822 (0.671–1.007)	0.059	0.823 (0.664–1.020)	0.075
Missing clinical values as categories	735	0.855 (0.685–1.066)	0.164	0.795 (0.633–0.998)	0.048
Number of comorbidities instead of Charlson component	681	0.813 (0.642–1.030)	0.086	0.765 (0.601–0.975)	0.030

(B) Low versus high educational attainment

Model	n	Topical/non-invasive vs standard excision, aOR (95% CI)	p	MMS vs standard excision, aOR (95% CI)	p
Primary model	681	0.123 (0.034–0.450)	0.002	0.533 (0.232–1.222)	0.137
Including "other" education	693	0.122 (0.033–0.446)	0.001	0.516 (0.224–1.187)	0.119
Country instead of center	681	0.129 (0.036–0.466)	0.002	0.546 (0.243–1.225)	0.142
Diagnosis instead of histologic group	696	0.122 (0.036–0.417)	<0.001	0.581 (0.267–1.266)	0.172
Missing clinical values as categories	735	0.194 (0.067–0.567)	0.003	0.545 (0.240–1.238)	0.147
Number of comorbidities instead of Charlson component	681	0.124 (0.034–0.452)	0.002	0.523 (0.228–1.202)	0.127

Age estimates are per 10-year increase. Standard surgical excision is the reference outcome. High education is the predictor reference. aOR, adjusted odds ratio; CI, confidence interval; MMS, Mohs micrographic surgery.

Table S4. Primary model fit and global likelihood-ratio tests (n = 681).

Model component	Likelihood-ratio test / fit statistic
Overall model	χ^2 = 613.650; 28 df; p < 0.001
McFadden pseudo-R^2	0.427
Age	χ^2 = 7.579; 2 df; p = 0.023
Sex	χ^2 = 5.519; 2 df; p = 0.063
Marital status	χ^2 = 0.068; 2 df; p = 0.967
Educational level	χ^2 = 16.995; 4 df; p = 0.002
Histologic group	χ^2 = 218.439; 6 df; p < 0.001
Tumor size	χ^2 = 22.117; 2 df; p < 0.001
Tumor location	χ^2 = 124.157; 2 df; p < 0.001
Recruiting center	χ^2 = 89.345; 6 df; p < 0.001
Comorbidity	χ^2 = 2.053; 2 df; p = 0.358
